# Why Muscle is an Efficient Shock Absorber

**DOI:** 10.1371/journal.pone.0085739

**Published:** 2014-01-23

**Authors:** Michael A. Ferenczi, Sergey Y. Bershitsky, Natalia A. Koubassova, Galina V. Kopylova, Manuel Fernandez, Theyencheri Narayanan, Andrey K. Tsaturyan

**Affiliations:** 1 National Heart and Lung Institute, Imperial College London, London, UK and Lee Kong Chian School of Medicine, Nanyang Technological University, Singapore, Singapore; 2 Institute of Immunology and Physiology, Ural Branch of the Russian Academy of Sciences, Yekaterinburg, Russia; 3 Institute of Mechanics, Lomonosov Moscow University, Moscow, Russia; 4 European Synchrotron Radiation Facility, Grenoble, France; University of Debrecen, Hungary

## Abstract

Skeletal muscles power body movement by converting free energy of ATP hydrolysis into mechanical work. During the landing phase of running or jumping some activated skeletal muscles are subjected to stretch. Upon stretch they absorb body energy quickly and effectively thus protecting joints and bones from impact damage. This is achieved because during lengthening, skeletal muscle bears higher force and has higher instantaneous stiffness than during isometric contraction, and yet consumes very little ATP. We wish to understand how the actomyosin molecules change their structure and interaction to implement these physiologically useful mechanical and thermodynamical properties. We monitored changes in the low angle x-ray diffraction pattern of rabbit skeletal muscle fibers during ramp stretch compared to those during isometric contraction at physiological temperature using synchrotron radiation. The intensities of the off-meridional layer lines and fine interference structure of the meridional M3 myosin x-ray reflection were resolved. Mechanical and structural data show that upon stretch the fraction of actin-bound myosin heads is higher than during isometric contraction. On the other hand, the intensities of the actin layer lines are lower than during isometric contraction. Taken together, these results suggest that during stretch, a significant fraction of actin-bound heads is bound non-stereo-specifically, i.e. they are disordered azimuthally although stiff axially. As the strong or stereo-specific myosin binding to actin is necessary for actin activation of the myosin ATPase, this finding explains the low metabolic cost of energy absorption by muscle during the landing phase of locomotion.

## Introduction

Apart from fast, efficient and coordinated contraction of several skeletal muscles, running and jumping require effective absorption of the kinetic energy of the body during the landing phase to cushion it and prevent injury. This is done not only by joints, bones and tendons, but also by activated muscles which resist stretch. Stretching a fully activated muscle fiber of the frog induces an increase in tension above its isometric level [1]. When stretch velocity reaches a certain value, tension saturates at a level higher than isometric which does not increase further upon further increase in stretch velocity [1]. This saturated tension level does not depend on temperature [2]. Instantaneous stiffness of contracting muscle also increases during stretch compared to its isometric level, thus providing effective resistance to fast stretching [1], [3]. This stiffness rise appears within a fraction of a millisecond after a step stretch [4]. During stretch the ATPase rate in muscle is very low [5], [6] so that energy is absorbed at no metabolic cost. External work is absorbed inside the muscle during stretch and is not converted into heat until stretch is finished [7]. Although non-actomyosin structures such as titin may contribute to the mechanical response of contracting muscle to stretch [8], it is generally believed that the mechanical and biochemical features mentioned above are mainly caused by the properties of actin-myosin cross-bridges, at least for stretches of moderate amplitude. Brunello *et al*. [9] suggested that during isometric contraction a majority of myosin molecules involved in actin binding have only one of their two globular heads bound to actin and that stretch causes fast binding of the second partner head to the closest actin monomer on the same thin filament ∼5.5 nm closer towards the sarcomere M-line. Correspondingly, the initially bound head is closer to the Z-disk than its newly bound partner. The attachment of the second head in a pair may explain the high instantaneous stiffness of muscle subjected to stretch and the apparent independence of tension on the stretch velocity. Indeed fast binding of the M-ward head followed by detachment of the Z-ward head, which is able to reattach quickly, could explain why force is independent of the stretch velocity. If the speed of the head detachment/attachment is high enough to provide reattachment of the second head while the first one remains attached to actin, one would expect force and stiffness, or the fraction of the actin bound heads, to remain high and to be velocity independent within a certain range of stretch velocity.

On the basis of measurement of the M3 meridional x-ray reflection, Brunello *et al*. [9] suggest that the catalytic domains of myosin heads bound to actin during and after a stretch have the same configuration as during isometric contraction, so that only their light chain domains bend upon strain. However this interpretation was not tested experimentally. To reveal the 3D structure of the actin-myosin complexes in muscle fibers under stretch, a detailed 2D diffraction pattern is required. Here we report the results of measuring changes in the 2D x-ray diffraction pattern induced by a ramp stretch of rabbit permeabilized muscle fibers contracting at near physiological temperature. The results support the view that stretch promotes binding of myosin heads to actin. In addition the data show that the catalytic domains of a majority of the heads bound to actin during muscle stretch have a wide range of azimuthal angles with respect to actin. Such attachment mode is known as non-stereo-specific binding [10], [11]. A locking transition of attached heads from a non-stereo-specific to a stereo-specific binding was suggested to be a part of the force-generating process. According to the ‘roll-and lock’ model, stretch prevents the transition to the locked state and keeps the heads non-stereo-specifically attached to actin, and yet contribute to muscle stiffness and therefore resist stretch [12]. We believe that this forced unlocking of stereo-specifically bound myosin heads and their transition to a non-stereo actin binding state promotes fast recruitment of previously detached heads. Thus the number of myosin heads which are bound to actin and resist stretch becomes higher than the number needed to produce and bear isometric force. The non-stereo-specific attachment of myosin heads during stretch explains very low ATPase rate in muscle subjected to stretch and other features of eccentrically contracting muscle.

## Materials and Methods

### Ethics statement, specimen preparation, solutions, experimental protocol

Muscle fibers were harvested from New Zealand exbreeder rabbits (5 kg) provided by a Home Office approved supplier, sacrificed by an intravenous overdose of sodium pentobarbital (100–200 mg/kg), followed by dislocation of the spinal cord in accordance with Home Office Schedule 1 Protocol (UK). Bundles of muscle fibers from *m. psoas* of the rabbit were prepared and stored in 50% glycerol relaxing solution at −20°C as described [13]. Thin bundles of 2–3 fibers were dissected and mounted into the experimental setup with one end attached with shellac glue to a force transducer and another to a length change motor [13]. A remote-controlled set-up [13] was placed on the sample table of the ID02 beam-line at the European Synchrotron Radiation Facility (ESRF, Grenoble, France) so that the fiber bundle was vertical with its motor end below the force transducer. When the beam-line hutch was interlocked, the bundle was transferred from the relaxing solution to the activating one containing 30 µM Ca^2+^at 0–2°C for 5–7 s and then to an air trough where its temperature increased to 4–5°C.

Relaxing solution contained (in mM): 3-(N-Morpholino)propanesulfonic acid (MOPS) 100; MgAcetate 7.5; Adenosine-5′-triphosphate (Na_2_ATP) 5, Ethylene glycol tetraacetic acid (EGTA) 5, Phosphocreatine (PCr) 22, DL-Dithiothreitol (DTT) 80, pH 7.1 at 20°C. Activating solution had the same composition except EGTA was substituted for CaEGTA and the PCr concentration was reduced to 20 mM. Mechanical and T-jump apparatus were described previously [11], [13].

**Figure 1:**
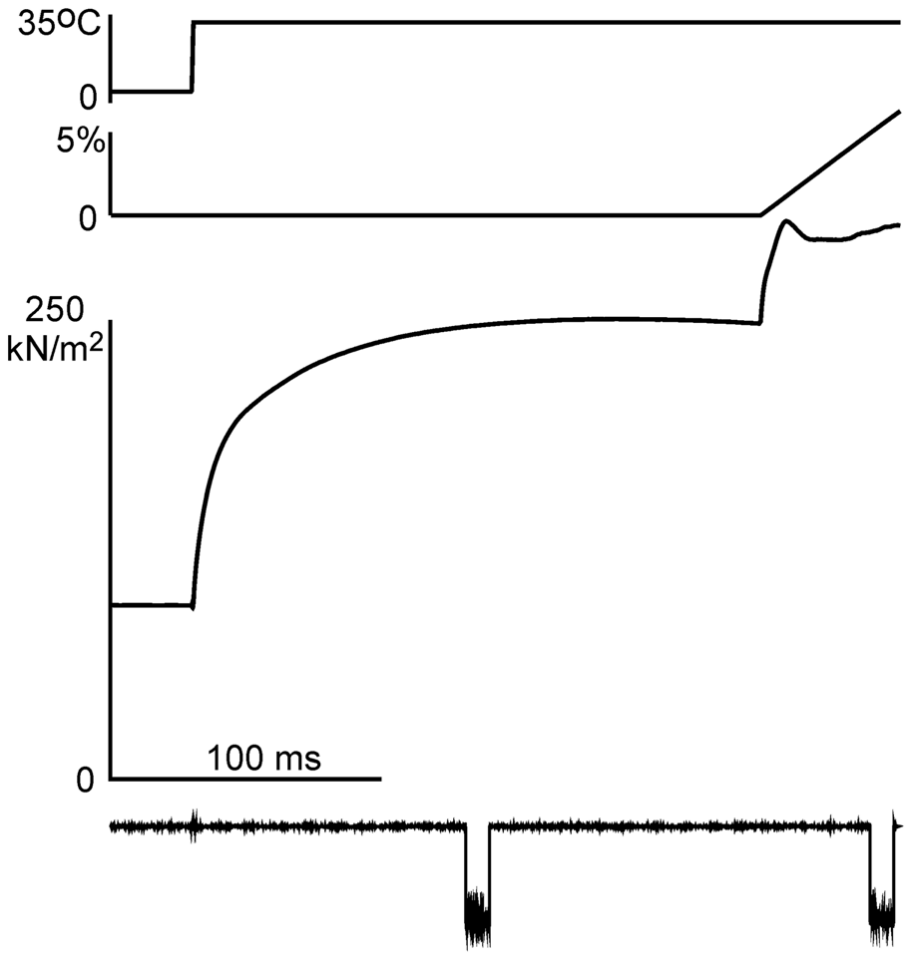
Experimental protocol. Averaged records (from top to bottom): calculated temperature, motor position (in % of bundle length), tension and an example of the x-ray exposure framing (signal from a pin diode in a run of the experimental protocol). Noise on the pin diode signal, 30 ms from the beginning of the recording is caused by the high voltage heating pulse of the T-jump apparatus.

In 2–3 s after the T-jump, a ramp stretch protocol was initiated and two x-ray diffraction patterns were recorded (Fig. 1). In the first series of experiments with short x-ray camera and rather long (10–30 ms) x-ray exposure up to 3 successful runs of the protocol were performed before a bundle broke or tension at the elevated temperature decreased to less than 85% of the value in the first run. A total of 9 successful runs of the protocol were achieved using 5 bundles. In the second series of experiments with a longer camera and short (5 ms) x-ray exposure, 8 to 12 successful runs of the protocol were performed for each of 3 bundles (31 runs in total). The x-ray diffraction patterns recorded during isometric contractions in each of two series were added together, and so were those recorded during stretch. The signal from the strong M3 reflection in the second series was good enough to calculate bundle-to-bundle statistics.

### X-ray diffraction

The x-ray beam of ∼0.1 nm wavelength was focused on the FReLoN CCD detector operating at 2048×256 (meridian × equator) pixels. Sample to detector distance was 3.0 m and 6.1 m for the first and second series of experiments, respectively. Diffraction patterns were corrected for dark current of the CCD, spatial homogeneity and distortion and then analyzed as described [14]. The beam size on the sample was 70×400 ***μ***m (vertical × horizontal full width at half-maximum, FWHM) with a flux of up to ∼3.8×10^13^ photons/s. The vertical FWHM of the beam image on the detector was 2.36 pixels. Prior to subtraction, the patterns were corrected for the precise exposure duration as measured with a pin diode signal (Fig. 1). To correct for a decrease in the bundle volume exposed to the x-rays during stretch the second pattern was multiplied by a factor dependent on the average elongation of the bundle in the middle of the 2nd frame (Fig. 1). The factor was 1.035–1.059 depending on the amount and duration of stretch and on duration of the x-ray frame. Then all isometric frames as well as the frames collected during stretch were added together, background was subtracted and the patterns were mirrored as described [14]. The meridional and off-meridional x-ray intensities were obtained using radial integration and background subtraction as described [14].

### Modeling x-ray diffraction on muscle

To model 2D x-ray diffraction patterns we used previously described model [14] with some modifications. Details of modeling of the interference splitting of the M3 meridional reflection was performed as described [15]. During isometric contraction 40% of the heads were assumed to be strongly (stereo-specifically) bound to actin by one head domain of a myosin molecule [14]. To check whether the stereo-specific model [9] can explain our data we assumed that upon stretch, half of these actin-bound myosin molecules bind neighbor actin monomer with their second partner head. Alternatively, we also checked the possibility that the 50% increase in the number of myosin heads is caused by stereo-specific binding of the heads of myosin molecules which were not interacting with actin under isometric conditions. The calculated intensity of the A1 layer line was very close to that after 50% binding of partner heads (data not shown). To simulate non-stereo-specific binding of myosin heads to actin during activation we assumed that heads which were bound to actin during isometric contraction unlock and adopt a wide range of azimuthal angles. The angle distribution was random in the range of 60° or 80°. The second partner head of a half of the actin-bound myosin molecules was also attached to the actin monomer that is 5.5 nm closer to the M-band than that occupied by the first head. Modeling of the M3 interference splitting was performed with a point diffractor model as described [15].

## Results and Discussion

### Experimental protocol

A bundle of 2–3 muscle fibers was transferred from a relaxing solution to a trough filled with cold (0–1°C) activating solution containing 30 ***μ***M Ca^2+^. After 5–7 seconds of activation the bundle was transferred to a cold empty trough where its temperature increased to 4–5°C. There the bundle was subjected to joule temperature jump (T-jump) to 31–34°C [13], [14]. When steady-state isometric tension was achieved, the bundle was exposed to x-rays for 5, 10, 20 or 30 ms depending on the x-ray flux (Fig. 1). Then it was stretched by 5–6% of its length in 40–50 ms at a constant velocity of 1.2–1.5 length/s. Another x-ray frame of the same duration was recorded just before the end of the stretch (Fig. 1). The change in average half-sarcomere length at the beginning of the second frame was at least 20 nm to ensure detachment of myosin heads prior to their reattachment to actin.

### Stiffness changes suggest that more myosin heads are bound to actin during stretch

**Figure 2:**
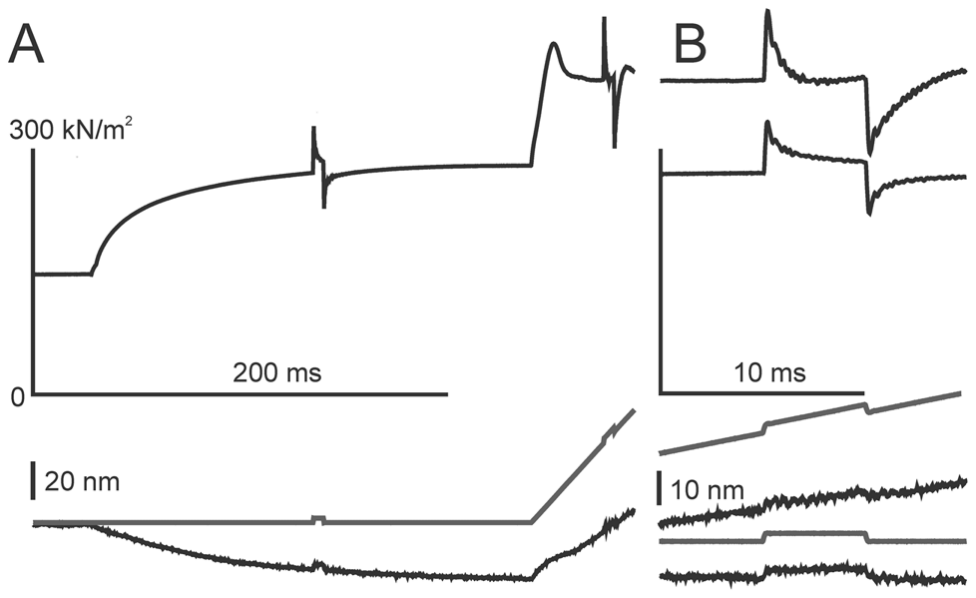
Change in instantaneous stiffness measured in a control experiment with a single muscle fiber. *A*: records from top to bottom: tension, change in half-sarcomere length, ΔSL, calculated from the motor position for SL = 2.45 ***μ***m; ΔSL measured by laser diffraction (more noisy traces). *B*: two fragments of the records shown in *A* on an expanded time scale to visualize changes in tension and sarcomere length during and after the step length changes applied during isometric contraction and ramp stretch. The length changes were used to measure fiber stiffness.

In control experiments with single fibers we measured instantaneous bundle stiffness with step length changes which were applied during the protocol in the time intervals which approximately correspond to the middles of the x-ray frames (Fig. 2). Changes in sarcomere length were monitored with a position sensitive photodiode [13]. Stretch led to a 33–35% increase in the instantaneous stiffness as found previously in intact frog muscle fibers at a lower temperature [1]. As about a half of the compliance of a half-sarcomere is due to extensibility of the thin and thick filaments [17], the stiffness data suggest that the fraction of myosin heads bound to actin doubled during the ramp stretch. The increase in instantaneous stiffness upon stretch found here is close to that reported earlier for transition of fully activated fast skeletal muscle fibers of the frog or the rabbit to rigor [18], [19]. As all myosin heads are strongly bound to actin in rigor [20], [21] and about 42% of them are strongly bound to actin during isometric contraction at 31–34°C [16] the data suggest that almost all heads become actin-bound during ramp stretch at a velocity of 1.4 ***μ***m/s per half-sarcomere or higher.

### Changes in the x-ray diffraction pattern

**Figure 3:**
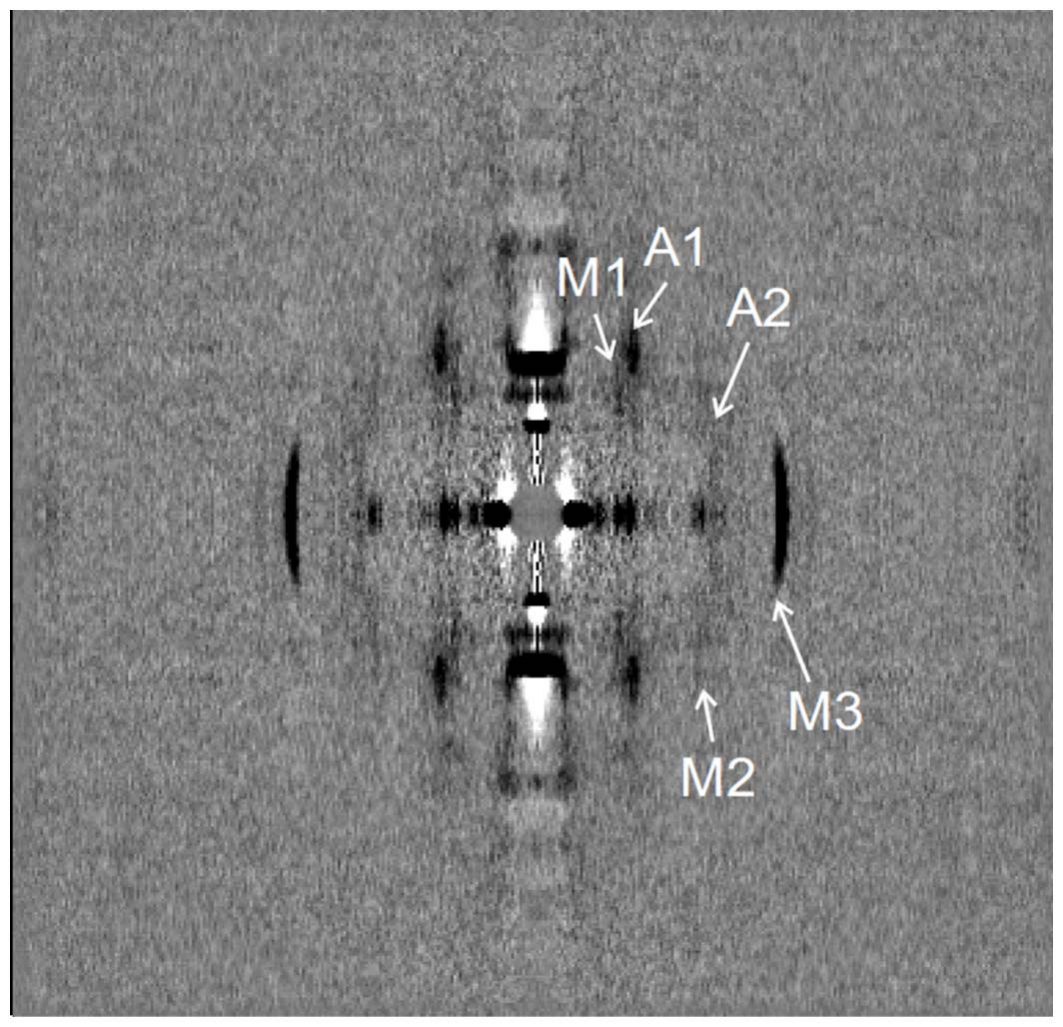
Low-angle X-ray diffraction. The difference between the diffraction patterns recorded during the isometric and the stretching phases, collected from 9 runs of the protocol in 5 bundles of muscle fibers in the first experimental series. The isometric pattern was subtracted from the pattern collected during stretch that had been multiplied by a factor of 1.035–1.059 to correct for the stretch-induced decrease in the fiber volume exposed to the x-rays. White and black correspond to an increase and decrease in the intensity during stretch compared to isometric contraction, respectively. X-ray reflections of interest are marked.

**Figure 4:**
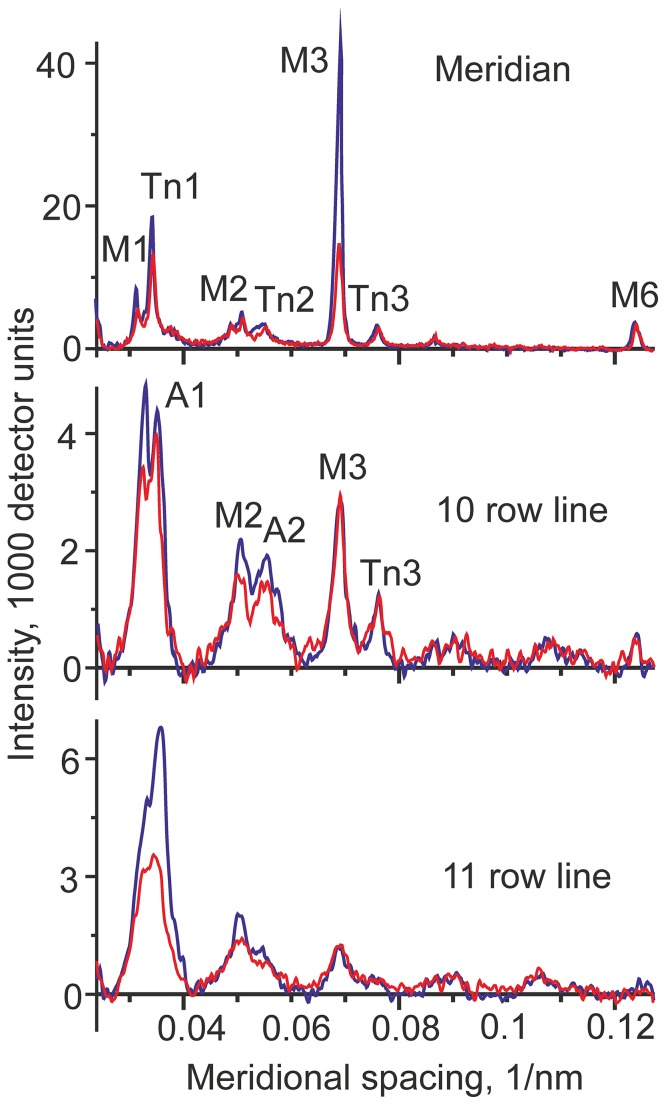
Meridional profiles of the meridional (top) and off-meridional intensities during isometric contraction (blue lines) and ramp stretch (red lines) in the 1^st^ series of experiments. The intensities were integrated in the reciprocal radii regions of ±0.018 nm^−1^ (meridian), 0.018–0.035 nm^−1^ (10 row line), and 0.035–0.06 nm^−1^ (11 row line) after correction for the change in specimen volume in the x-ray beam and mirroring four quadrants of the x-ray diffraction pattern. Background was subtracted as described [11], [14]. The positions of some X-ray reflections of interest are marked.

The difference between the diffraction patterns collected during isometric contraction and during stretch from all 9 runs of the protocol in the first series of experiments is shown in Fig. 3. Meridional intensity profiles at different reciprocal radii during isometric contraction and stretch are shown in Fig. 4. The main changes in the diffraction pattern caused by the ramp stretch are summarized in Table 1. The most prominent changes were as follows: decreases in the meridional intensity of the M3 myosin reflection at ∼(14.5 nm)^−1^ in the off-meridional intensity of the 1st off-meridional myosin layer line (M1, spacing of ∼(43 nm)^−1^) and of the 1st actin layer line (A1, spacing of ∼(36 nm)^−1^). The decrease in the intensities of the actin and myosin layer lines upon stretch was also seen in the 2nd order myosin and actin layer lines, M2 and A2 (Figs. 3,4). Changes in the intensities of the M1 and M3 myosin reflections upon stretch were similar to those observed in whole frog skeletal muscle at low temperature [22], [23]. Changes in the intensity of actin layer lines during stretch have never been reported previously.

**Table 1:** Changes in the intensity and spacing of some x-ray reflections upon stretch.

State	*I*_M1_ 1, %	*I*_A1_ 1, %	*I*_M3_ 2, %	*S*_M3_ 3, nm	*S*_M3_*^4^, nm	*R*_M3_ 5, Mean±SD, (n)
Isometric	1*00*	*100*	*100*	14.562	14.570	0.36±0.03 (6)
Stretch	74	62	39	14.577	14.582	0.65±0.06 (6)

1 The off-meridional x-ray intensities of the M1 and A1 layer lines in the region of the 10 and 11 row lines (0.018–0.06 nm^−1^).

2 The meridional x-ray intensity of the M3 reflection integrated in the radial range of ±0.018 nm^−1^ and meridional range of 0.066–0.072 nm^−1^ in the experiments with short x-ray camera.

3 Average meridional spacing of the M3 reflection measured as the center of gravity of the intensity profile in the same meridional and radial range as *I*_M3_. Summed data for all three experiments with long x-ray camera.

4 Average spacing of the M3 reflection calculated only for the data points where the intensity was above 5% of the peak value to avoid possible errors caused by background subtraction or the presence of non-myosin reflections; the same data set as for *S*_M3_.

5 The ratio of the amplitudes of the high- and low-angle peaks in the fine structure of the M3 reflection. Statistics was obtained by analysing the M3 intensities in each half of the pattern in three experiments with long x-ray camera.

### A decrease in the intensities of myosin layer lines, M1 and M2, confirms that stretch recruits detached myosin heads into actin binding

Upon stretch the intensities of the myosin layer lines M1 and M2 decreased by 25–30% (Figs. 3,4) suggesting that fewer myosin heads are incorporated into the myosin-based helix of the thick filaments. As the M1 and M2 intensities mainly originate from detached myosin heads [10] and their intensities are maximal in the relaxed state where all myosin heads are detached from actin, the data suggest that more myosin heads become bound to actin during the stretch compared to isometric contraction. Judging from the stiffness rise (Fig. 2) and the decrease in the M1 intensity (Figs. 3,4) we estimate ∼50% increase in fraction of myosin heads bound to actin during stretch in a fast mammalian muscle at near-physiological temperature. Similar estimates were obtained by Brunello *et al*. [9] in experiments with step stretches in intact frog muscle fibers.

Our data does not distinguish between the possibilities that the increase in the number of myosin heads bound to actin is caused by binding of the second partner head of the same myosin molecule or by binding of the heads whose partner heads were also detached from actin during isometric contraction. However as the increase in stiffness upon stretch was shown to occur very quickly, in a fraction of millisecond after a step stretch [4], i.e. much faster than attachment of myosin heads during activation or force redevelopment at a constant length, the assumption that the fast binding of detached heads is promoted by the preceding movement of their partner heads already attached to actin is attractive.

### A decrease in the intensities of the actin layer lines, A1 and A2, suggests that during stretch myosin heads are bound to actin non-stereo-specifically

**Figure 5:**
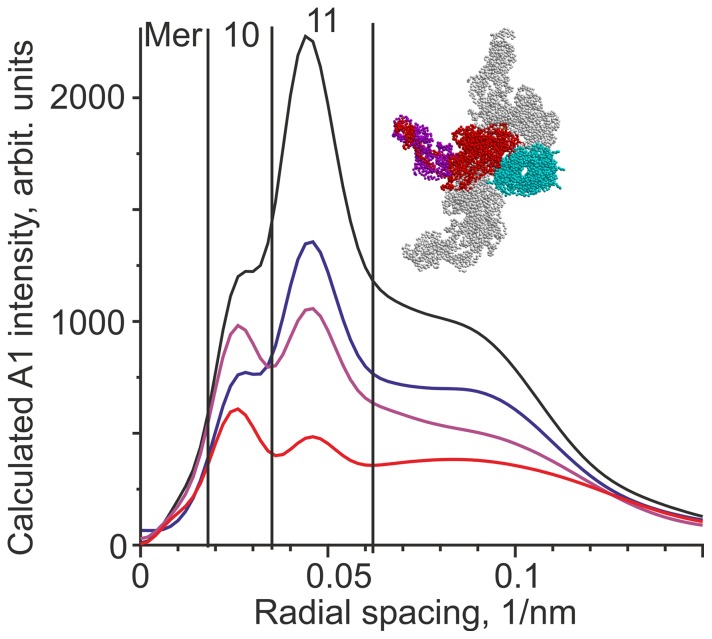
Calculated intensity of the A1 actin layer line. The blue line corresponds to 40% of the total number of myosin heads bound to actin stereo-specifically by only one of the two heads of a myosin molecule. The black line corresponds to 60% of myosin heads stereo-specifically bound to actin: 20% of myosin molecules with one head only and the other 20% with both their heads. The purple and red lines correspond to non-stereo-specific attachment of the same 60% of heads with random uniform distribution of the azimuthal orientation angles within ranges of 60° or 80°, respectively. The vertical lines show the integration ranges for meridian (Mer), and the 10 and 11 row lines used for the experimental data shown in Fig. 3. Inset shows an actin filament (cyan, viewed along the filament axis) with a pair of stereo-specifically bound myosin heads (red heavy chains, magenta light chains). The same pair rotated by ±60° is shown in gray.

The most striking feature of the change in the x-ray diffraction pattern during a ramp stretch is a significant decrease in the intensities of the actin layer lines A1 and A2 compared to their value during isometric contraction (Fig. 3). Brunello *et al*. [9] assumed that catalytic domains of all actin-bound myosin heads are in the same stereo-specific configuration with respect to actin. According to their interpretation, which is in the framework of a conventional ‘lever arm model’ [24], [25], the power stroke or stretching force is only induced by a tilt of the lever arm, i.e. the light chain domain of the head. As stretch leads to an increase in the fraction of actin-bound myosin heads, the A1 intensity is expected to rise significantly. Indeed, calculation using a model described previously [14], [16] for a 50% increase in the fraction of actin bound heads predicts a two-fold increase in the A1 intensity compared to its isometric level (Fig. 5). The result does not depend on which myosin head bind actin, whether they are those whose partner head is already bound during isometric contraction or those which had both heads detached before stretch (data not shown). Besides, the A1 intensity was shown not to be affected by the tilt of the lever arm, if the number of stereo-specific myosin heads remains constant [16].

These calculations indicate that the increase in the number of actin-bound heads and the simultaneous decrease in the A1 intensity upon stretch can only be explained if stretch induces an unlocking of stereo-specifically bound heads as was suggested by our ‘roll and lock’ model [12]. The newly formed heads also do not bind actin in a strong, stereo-specific manner, because if they did, the A1 intensity would rise correspondingly. The term ‘non-stereo-specific’ means that the catalytic domains of the heads bound to actin have a wide range of azimuthal (and possibly axial) angles with respect to the actin monomers they are bound to [11]. We simulated the observed decrease in the A1 intensity quantitatively (Fig. 5), assuming that a stretch increases the fraction of actin-bound heads from 40% to 60%, and that all bound heads have a random azimuthal disorder with respect to actin in a ±60–80° range.

### Changes in fine structure of the M3 myosin meridional reflection suggest a ∼1 nm axial displacement of myosin heads upon stretch

**Figure 6:**
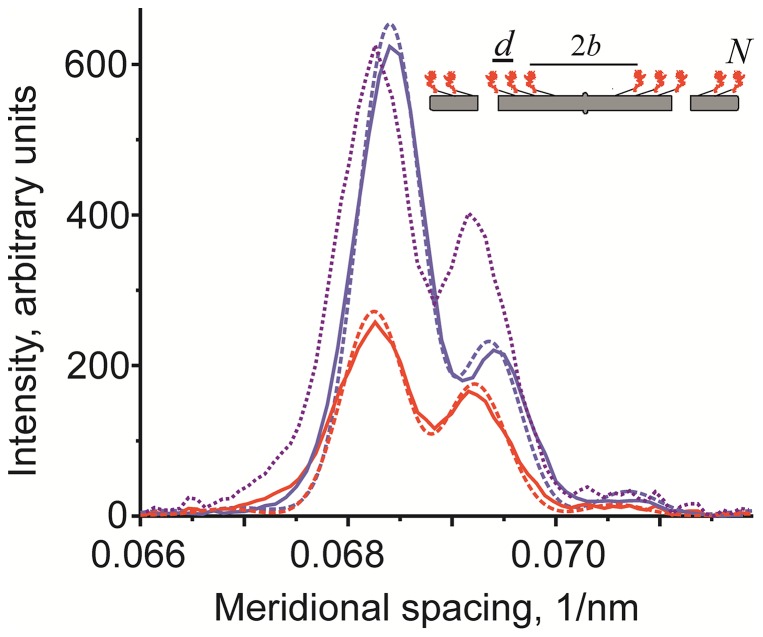
M3 profiles during isometric contraction (blue continuous line) and stretch (red continuous line) in the second series of experiments with a long x-ray camera averaged over two halves of the patterns and 32 runs of the protocol in three fiber bundles. The M3 profile during stretch scaled to equalize its low-angle peak with that measured during isometric contraction is shown by the dotted magenta line. Results of the modeling of the M3 meridional profile using the point diffractor model [15] with a half-bare zone length *b*, with the distance between crowns of myosin heads *d* and the total number of crowns *N* = 50 in each half of a thick filament: *b* = 74.75 nm and *d* = 14.564 nm that provide the best fit to the isometric data (blue dashed line) and *b* = 75.8 nm, *d* = 14.569 nm giving the best fit to the data obtained during stretch (red dashed line). The calculated profiles were then convoluted with a Gaussian point spread function with the standard deviation of 2.26 nm^−1^.

Averaged profiles of the M3 myosin meridional reflection are shown in Fig. 6. Stretch induced not only a decrease in the M3 intensity, but also a change in its average spacing and in the ratio of the high- and low-angle peaks in its interference splitting (Fig. 6, Table 1). The splitting is caused by interference of x-rays scattered by two halves of a sarcomere [26], [27]. The ratio of the peaks is very sensitive to small axial movements of myosin heads [28]. The average spacing of the M3 reflection measured as the center of gravity of the peak increased during stretch by 0.1%. The ratio *R* of the intensity of the high- and low-angle peaks in the fine structure of the M3 reflection increased from 0.36 to 0.65 (Table 1). Fitting the data with a point diffractor model [15], (Fig. 6) suggests that observed changes in the fine structure of the M3 reflection can be explained by a 1.05 nm axial movement of diffracting mass towards the Z-disk and by a shift in the axial distance between the crowns of myosin heads from 14.564 nm during isometric contraction to 14.569 nm upon stretch (Fig. 6).

### A scheme of structural transition in the actin-myosin complexes upon muscle stretch

**Figure 7:**
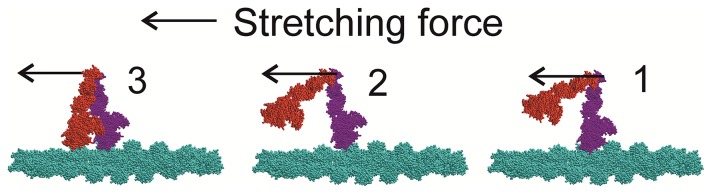
Schematic model of the movement of myosin heads upon muscle stretch that explains our data. During isometric contraction (state 1) only one (purple) head of a majority of myosin molecules is stereo-specifically bound to actin (cyan). Stretch unlocks the bound head to a non-stereo-specifically attached state and brings the distal part of the partner head to a position from which is can easily bind to a neighbor actin monomer (state 2). Then the second (red) head quickly binds actin also non-stereo-specifically (state 3). As both heads are bound non-stereo-specifically, the A1 intensity is low and stiffness is high. Further stretch leads to detachment of the purple head followed by its rapid rebinding to an actin 5.5 nm closer to the M-line of a sarcomere, thus producing a ‘head over head’ walking. The M-line of the sarcomere is on the left and the Z-disk is on the right.

A schematic model that explains our findings as well as data of Brunello *et al*. [9] and recent ATPase measurements [6] is shown in Fig. 7. During isometric contraction only one head (purple) of a myosin molecule is bound to actin while another is subjected to Brownian motion and therefore contributes very little to the diffraction pattern (Fig. 7, state 1). Stretch causes unlocking of the bound heads to a non-stereo-specifically bound state with a wide range of azimuthal orientations with respect to actin as predicted by the ‘roll and lock’ model [11]. The unlocking of one (purple) head brings its partner (red) head to a position from which it can easily attach to the next actin monomer, 5.5 nm closer to the M-line of a sarcomere (Fig. 7, state 2). As binding of the second head is also non-stereo-specific (Fig. 7, state 3) the A1 intensity remains low despite the increased number of actin-attached heads. Further stretch leads to detachment of the rear (purple) head followed by its fast reattachment to a next actin monomer proving a ‘head over head’ walking with a high fraction of actin-attached heads while only very few of them are bound stereo-specifically and consume ATP. This explains the low ATPase rate in contracting muscle during stretch [6] as the release of inorganic phosphate that is the rate limiting step for myosin ATPase requires stereo-specific binding of myosin head to actin [29].

We conclude that muscle stretch recruits more myosin heads into actin binding as was assumed previously [9]. Besides during stretch myosin heads become bound to actin non-stereo-specifically as evidenced by the decrease in the intensities of the actin layer lines, that is myosin heads are bound and disordered. There are two processes which provide fast absorption of kinetic energy of the body during the landing phase of running and jumping that protect bones and joints from damage without a metabolic cost: unlocking of strongly bound heads to a non-stereospecific state and recruitment of detached heads to non-stereo-specific binding to actin.
